# Copper adorned magnetic nanoparticles as a heterogeneous catalyst for Sonogashira coupling reaction in aqueous media

**DOI:** 10.1038/s41598-022-22567-5

**Published:** 2022-10-26

**Authors:** Safoora Sheikh, Mohammad Ali Nasseri, Ali Allahresani, Rajender S. Varma

**Affiliations:** 1 Department of Chemistry, Faculty of Basic Sciences, University of Birjand, P. O. Box 97175-615, Birjand, Iran; 2 Institut Für Organische Chemie, Universität Regensburg, Universitätsstr. 31, 93053 Regensburg, Germany; 3 Regional Centre of Advanced Technologies and Materials, Czech Advanced Technology and Research Institute, Palacký University in Olomouc, ˇSlechtitelů 27, 783 71 Olomouc, Czech Republic

**Keywords:** Chemistry, Nanoscience and technology

## Abstract

A nanomagnetic hydrophilic heterogeneous copper catalyst, termed γ-Fe_2_O_3_@PEG@PAMAM G_0_-Cu, has been successfully prepared and characterized using FT–IR, XRD, FE-SEM, TEM, EDX, mapping, TGA/DTG, VSM and ICP analyses. The catalyst displayed excellent activity for the palladium-free Sonogashira cross coupling reaction of various aryl iodides and bromides with phenylacetylene derivatives in pure water. The presence of polyethylene glycol coupled with hydrophilic character of the Cu-catalyst adorned on γ-Fe_2_O_3_ MNPs provides the ready dispersion of the catalyst particles in water, leading to higher catalytic performance as well as facile catalyst recovery via simple magnetic decantation. The recovered catalyst was reused for at least six successive runs with little reduction in its catalytic activity and any noticeable changes in its structure. The use of water as a green solvent, without requiring any additive or organic solvent, as well as the exploitation of abundant and low-cost copper catalyst instead of expensive Pd catalyst along with the catalyst recovery and scalability, make this method favorable from environmental and economic points of view for the Sonogashira coupling reaction.

## Introduction

Green chemistry entails the design for eco-friendly methods that at its core consider health and safety in line with the environmental preservation and economic aspects^1,2^. The 12 principles of green chemistry comprise, among others, the optimal use of energy, reduce or eliminate hazardous processes, lessening waste while promoting the use of renewable resources^3^. So, considering the vital role of chemistry in human life, one of the main objectives is to eliminate environmental hazardous parameters in chemical reactions via greener processes^4^. The promotion of catalytic protocols in aqueous solvents—instead of toxic and dangerous solvents—or the deployment of heterogeneous/recoverable catalysts, to a large extent, are considered measures of environmental friendliness in the chemical reactions^5^. Among suitable platforms, magnetic nanoparticles have garnered attention due to their ease of preparation, high active surface area and easy surface modification as stabilizing surfaces for homogeneous catalysts^6–8^; magnetic nature of nanoparticles is a simple way to recycle these catalysts deploying an external magnet. This separation method does not require much time and energy compared to centrifugation and filtration methods thus preventing the catalyst from being wasted during the separation process while enhancing the purity of products and reducing costs. Among magnetic nanoparticles, γ-Fe_2_O_3_ is one of the most widely used metal oxides which are often utilized in the preparation of heterogeneous catalysts^9^.

On the other hand, considering advantages of water as a green solvent, which is non-toxic, non-flammable, inexpensive and widely available, it can be a good alternative to toxic organic solvents^10^. Therefore, the design of efficient catalytic systems for organic synthesis in aqueous media is one of the sustainable means to adhere to the tenets of green chemistry^11–13^. However, often due to the insolubility or low solubility of the reaction materials in water, its use as a solvent has been limited and often necessitates the use additives such as surfactants, or auxiliary solvents for the enhancement of the reaction^14^. A desirable strategy that avoids the use of these additives is the deployment of hydrophilic catalysts that are well dispersed in the reaction medium. Various methods for the synthesis of hydrophilic catalysts have been proposed so far^15^ including adorning solid catalysts encompassing magnetic core with hydrophilic compounds, which can be a powerful tool in the synthesis of water dispersible metal complexes^16^.

Therefore, in view of the numerous favorable attributes of aforementioned magnetic nanoparticles (MNPs), modifying their surface to render them water dispersible is a key strategy; assorted hydrophilic materials such as cellulose, chitosan, polyethylene glycol have been explored earlier^17–20^.

The formation of carbon–carbon bonds in cross-coupling reactions is considered as one of the most important organic chemistry reactions, which have been extremely practical and useful tools for the formation of complex molecules from simple molecules^21–24^. Asymmetric carbon–carbon coupling reactions are equally important and valuable in the synthesis of natural and medicinal compounds^25^. The most prominent and well-recognized transverse couplings are Heck, Negishi, Suzuki and Sonogashira reactions^26^. The importance and superiority of this group of reactions in organic chemistry is affirmed by the award of the Nobel Prize in Chemistry in 2010 to Professors Heck, Negishi and Suzuki^27^ Sonogashira reaction was first reported by Sonogashira research group using Pd^0^/Cu^I^-catalyst, in 1975^28^.

So far, various and powerful catalysts have been designed and synthesized to promote the Sonogashira cross-coupling reaction^29–31^. However, the copper complexes are often deployed to catalyze this reaction and most of these complexes are insoluble in water^32^. Therefore, the design of active copper catalysts usable in water as a green and abundant solvent, is still challenging^15^.

**Figure 1:**
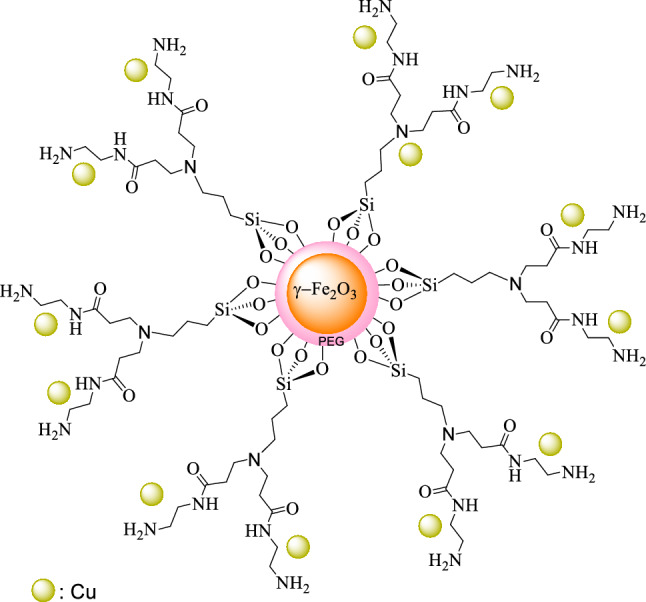
The proposed structure of γ-Fe_2_O_3_@PEG@PAMAM G_0_-Cu.

**Figure 2:**
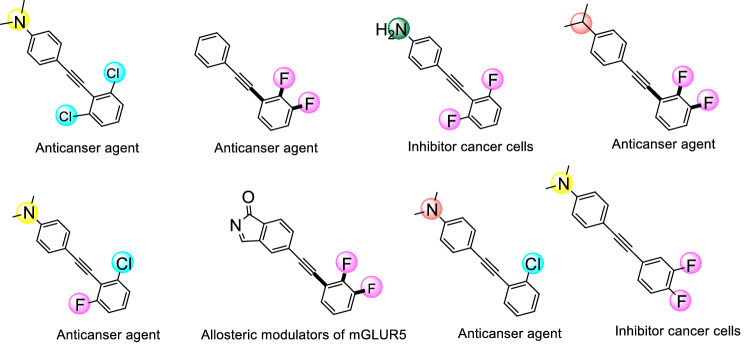
A few representative pharmacologically active compounds containing diphenylacetylene moieties.

Herein, as a part of our ongoing efforts to develop green and water dispersible catalysts, we describe the assembly of a copper-PAMAM dendrimer G_0_ catalyst anchored on polyethylene glycol coated γ-Fe_2_O_3_, through the siloxy linker, γ-Fe_2_O_3_@PEG@PAMAM G_0_-Cu (Fig. 1) and exemplified its performance and utility in the Sonogashira cross coupling as the target reaction, because diphenylacetylene derivatives are the precursors of drugs and biological substances and found in a wide variety of natural products and pharmaceutical agents (Fig. 2)^25^.

## Experimental section

### Preparation of γ-Fe_2_O_3_@PEG@PAMAM-Cu MNPs

**Figure 3:**
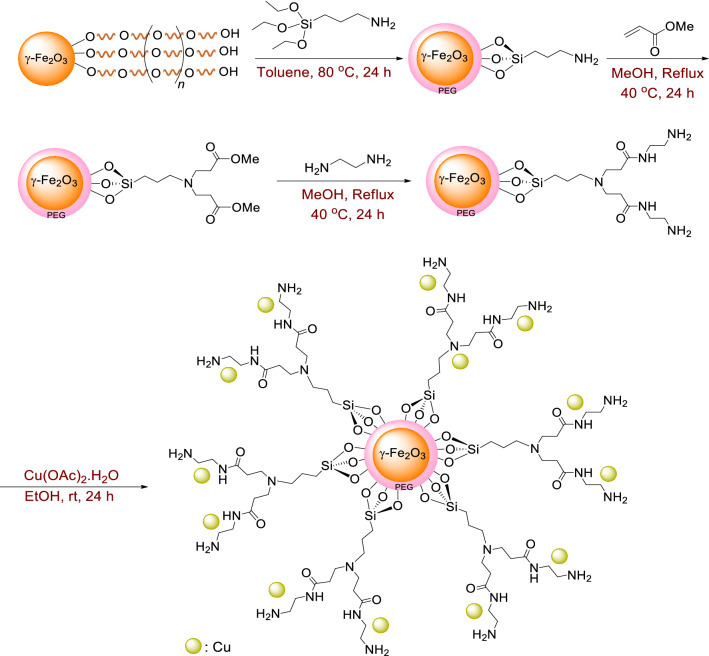
Preparation route for the catalyst, γ-Fe_2_O_3_@PEG@PAMAM G_0_-Cu.

The various steps of catalyst preparation are depicted in Fig. 3. Initially, γ–Fe_2_O_3_ MNPs were synthesized via co-precipitation method as reported in the literature^33^. Subsequently, γ-Fe_2_O_3_@PEG MNPs were prepared using the previous described method^34^.

To obtain γ-Fe_2_O_3_@PEG@APTES, γ-Fe_2_O_3_@PEG MNPs (1.0 g) was dispersed by sonication in dry toluene (30 mL) for 30 min. Then, (3-aminopropyl)triethoxysilane (APTES, 0.885 g, 0.83 mL, 4.0 mmol) was added dropwise to resulting dispersed solution of γ-Fe_2_O_3_@PEG MNPs, while the reaction was stirred under N_2_, at 25 °C. Then, the temprature was raised to reflux conditions and heating continued for 24 h. After completion of the reaction, the solid product was separated from the solvent using an external magnet and washed twice with anhydrous toluene and diethyl ether and then dried under vacuum 50 °C overnight, to obtain γ-Fe_2_O_3_@PEG@APTES (1.12 g).

Subsequently, methyl acrylate (MAc, 7 mL, 6.6 g) was added dropwise to mixture of γ-Fe_2_O_3_@PEG@APTES (1.0 g) dispersed in ethanol (30 mL), while the reaction was stirred under N_2_ atmosphere, at 25 °C. In the next step, the mixture was stirred and heated at 40 °C and the reaction continued for 24 h under N_2_. After completion of the reaction, solid product was separated by an external magnet and washed with ethanol three times and followed by vacuum drying at 50 °C overnight, to acquire γ-Fe_2_O_3_@PEG@MAc (1.21 g).

Subsequently, the γ-Fe_2_O_3_@PEG@PAMAM G_0_, was prepared by dispersion of 1 g of γ-Fe_2_O_3_@PEG@MAc in ethanol (40 mL) via sonication for 30 min, followed by addition of ethylenediamine (EDA, 0.1 mmol, 6.3 g) dissolved in ethanol (30 mL), while the reaction was being stirred under N_2_, at 25 °C. Then, the temprature was raised to 50 °C and continued the reaction for 48 h. The ensued solid product was separated by an external magnet, washed with ethanol three times, and finally dried at 50 °C overnight to obtain γ-Fe_2_O_3_@PEG@PAMAM (1.09 gr).

Finally, the earlier prepared γ-Fe_2_O_3_@PEG@PAMAM MNPs (1 g) was dispersed in ethanol (30 mL) for 30 min, and then, Cu(OAc)_2_·H_2_O (0.199 g, 1 mmol) dissolved in ethanol (20 mL) was added to ethanolic dispersed mixture, while the reaction was stirred at 50 °C. Then, the reaction was allowed to proceed for 24 h. Finally, γ-Fe_2_O_3_@PEG@PAMAM-Cu (1.08 g) was obtained following the sequence of separation by an external magnet, washing with ethanol and after drying the particles in vacuum at 50 °C overnight (Fig. 3). ICP-OES analysis showed presence 0.96 mmol of Cu on 1 gr of γ-Fe_2_O_3_@PEG@PAMAM G_0_-Cu (0.96 mmol, 0.06 gr, 0.25 wt%)).

### General Procedure for the Sonogashira cross coupling reaction (1a-p)

γ-Fe_2_O_3_@PEG@PAMAM G_0_-Cu (0.8 mol%, 0.006 g) was added to a mixture of NaOH (2.0 mmol), phenylacetylene (1.5 mmol), and aryl halide (1.0 mmol) in water (5 mL), under stirring in air and heated at 80 °C. The progress of reaction was monitored by TLC until completion. After the completion of the reaction, the nanocatalyst was separated by an external magnet and washed with ethyl acetate (3 × 3 mL). The desired product (liquid phase) was extracted with ethyl acetate (3 × 6 mL), and the organic layers were dried over anhydrous Na_2_SO_4_. After evaporation of the solvent, crude product was purified by column or flash chromatography to afford the pure products **1a**-**p**.

## Results and discussion

### Preparation of γ-Fe_2_O_3_@PEG@PAMAM G_0_-Cu

**Figure 4:**
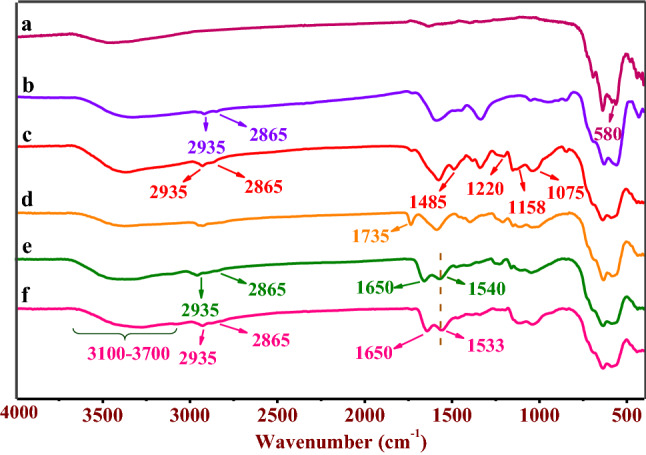
FT–IR spectra of the (**a**) γ–Fe_2_O_3_, (**b**) γ-Fe_2_O_3_@PEG, (**c**) γ-Fe_2_O_3_@PEG@APTES, (**d**) γ–Fe_2_O_3_@PEG@MAc, (**e**) γ-Fe_2_O_3_@PEG@PAMAM G_0_, (**f**) γ-Fe_2_O_3_@PEG@PAMAM G_0_-Cu.

**Figure 5:**
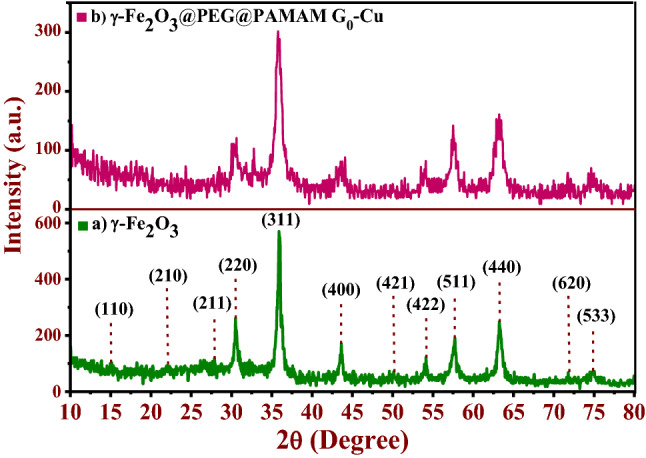
XRD patterns of (**a**) γ*‒*Fe_2_O_3_, and (**b**) γ-Fe_2_O_3_@PEG@PAMAM G_0_-Cu catalyst.

**Figure 6:**
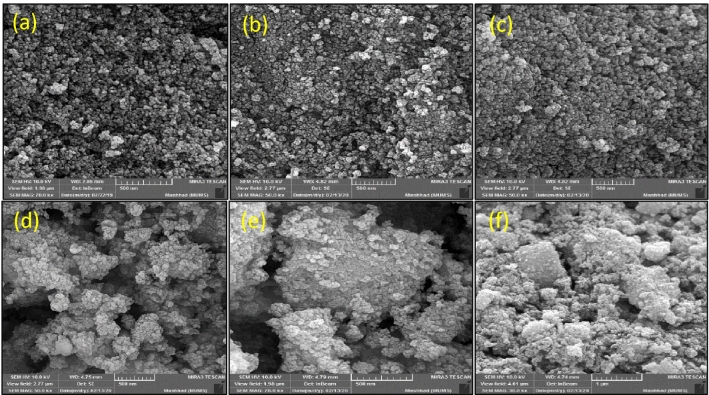
Field emission scanning electron microscopy pictures of the (**a**) γ–Fe_2_O_3_, (b) γ-Fe_2_O_3_@PEG, (**c**) γ-Fe_2_O_3_@PEG@APTES, (**d**) γ-Fe_2_O_3_@PEG@MAc, (**e**) γ-Fe_2_O_3_@PEG@PAMAM G_0_, and (**f**) γ-Fe_2_O_3_@PEG@PAMAM G_0_-Cu.

**Figure 7:**
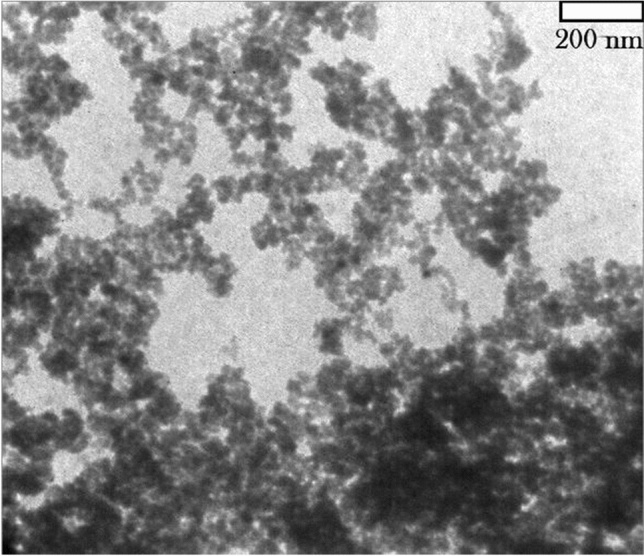
Transmission electron microscopy (TEM) picture of γ-Fe_2_O_3_@PEG@PAMAM G_0_-Cu catalyst.

**Figure 8:**
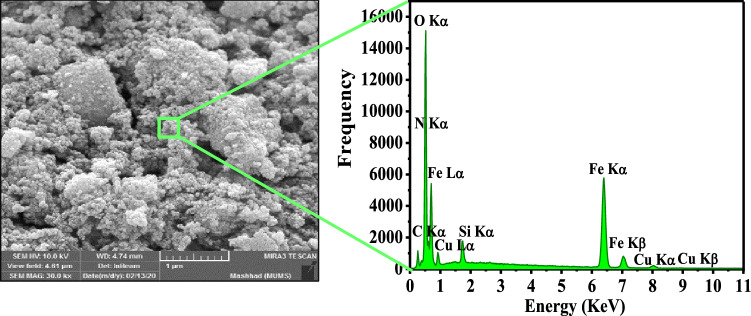
EDX analysis of the γ-Fe_2_O_3_@PEG@PAMAM G_0_-Cu catalyst.

**Figure 9:**
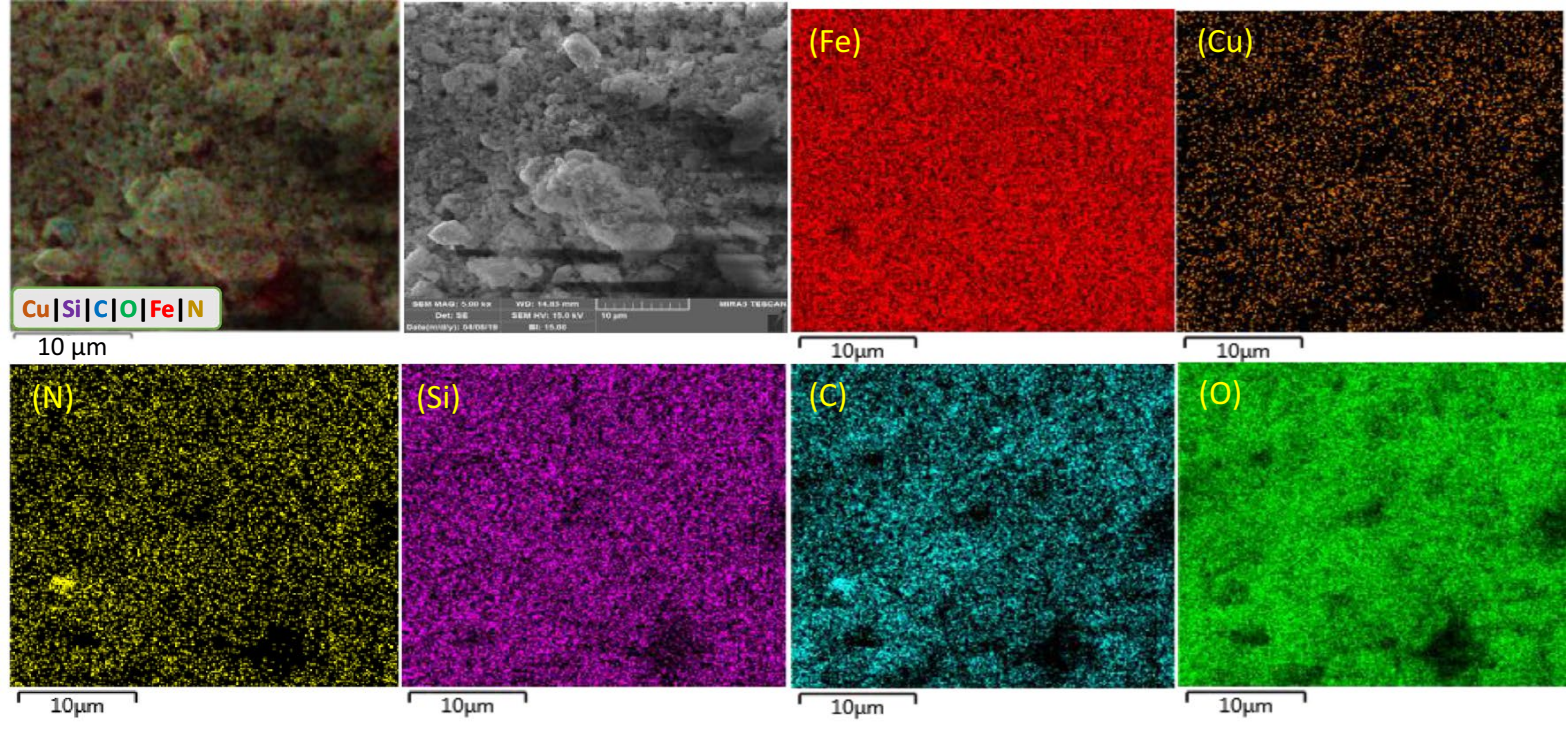
EDS elemental mappings of the γ-Fe_2_O_3_@PEG@PAMAM G_0_-Cu catalyst.

**Figure 10:**
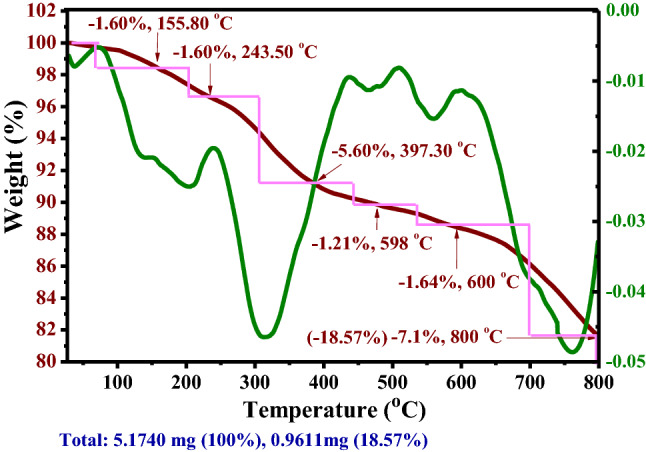
TGA/DTG/DTA weight loss curves of the γ-Fe_2_O_3_@PEG@PAMAM G_0_-Cu.

**Figure 11:**
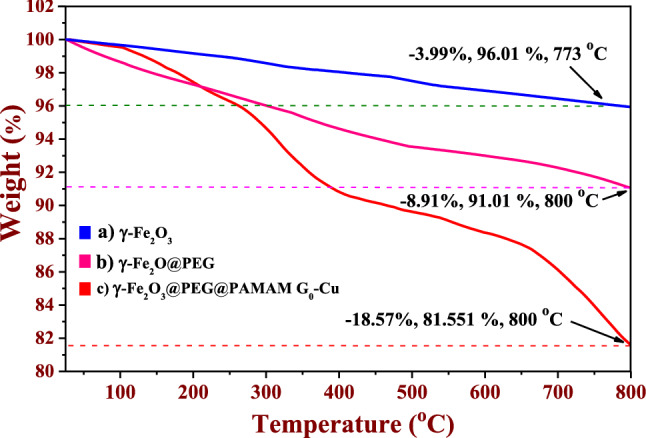
TGA weight loss curves of the (**a**) γ-Fe_2_O_3_ (**b**) γ-Fe_2_O_3_@PEG (**c**) γ-Fe_2_O_3_@PEG@PAMAM G_0_-Cu.

**Figure 12:**
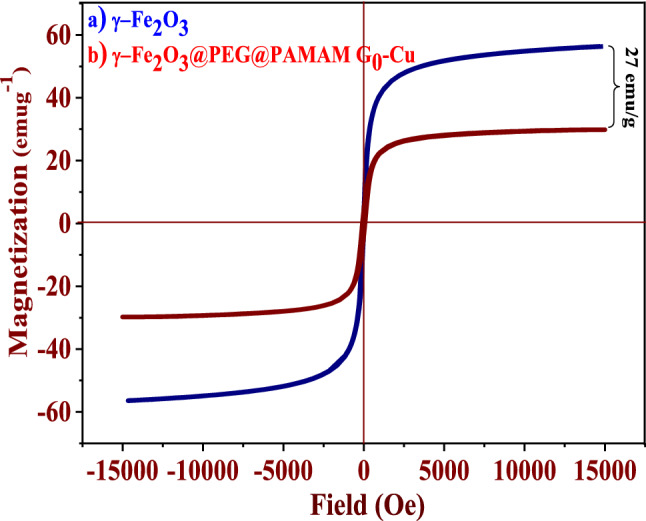
Magnetization curves of (**a**) γ-Fe_2_O_3_, (**b**) γ-Fe_2_O_3_@PEG@PAMAM G_0_-Cu at 300 K.

Initially, we selected γ-Fe_2_O_3_ MNPs as an attractive platform with unique features for the synthesis organic–inorganic heterogeneous catalyst. The preparation of γ-Fe_2_O_3_@PEG@PAMAM G_0_-Cu as a water-soluble catalyst was pursued starting from divergent synthesis of PAMAM dendrimer zero generation using amino siloxy linker immobilized on the surface γ-Fe_2_O_3_@PEG MNPs, as the bivalence core (–NH_2_). Eventually, deployment of Cu(OAc)_2_·H_2_O, resulted in the preparation of the γ-Fe_2_O_3_@PEG@PAMAM G_0_-Cu (Fig. 1). The catalyst structure was characterized via Fourier-transform infrared spectroscopy (FT-IR), transmission electron microscopy (TEM), X-ray diffraction analysis (XRD), field emission scanning electron microscopy (FE-SEM), inductively coupled plasma mass spectrometry (ICP), thermogravimetric analysis (TGA/DTG), value-stream mapping (VSM), MAP, nuclear magnetic resonance spectroscopy (NMR), and energy dispersive X-ray spectroscopy (EDX) (Figs. 4, 5, 6, 7, 8, 9, 10, 11, 12).

### Characterization of the γ-Fe_2_O_3_@PEG@PAMAM G_0_-Cu

Figure 4 presents the FT-IR spectra of the various synthetic segments of γ-Fe_2_O_3_@PEG@PAMAM G_0_-Cu, comprising, γ-Fe_2_O_3_ (a), γ-Fe_2_O_3_@PEG (b), γ-Fe_2_O_3_@PEG@APTES (c), γ-Fe_2_O_3_@PEG@MAc (d), γ-Fe_2_O_3_@PEG@ PAMAM G_0_ and (e), γ-Fe_2_O_3_@PEG@PAMAM G_0_-Cu (f). The strong absorption bond at about 580 cm^−1^ is assigned to the stretching vibrations of Fe–O in γ-Fe_2_O_3_ (Fig. 4a)^35,36^. The peak ~ 1075 cm^‒1^ is attributed to the stretching of C–O bond of PEG (Fig. 4b)^37^. The absorption bands at 1055 and 1158 cm^−1^ are ascribed to the stretching vibrations of Si–O which demonstrates the appropriate functionalization of γ-Fe_2_O_3_ by APTES (Fig. 4b)^38,39^. The peak at 1735 cm^−1^ illustrates the stretching vibrations of the carbonyl group (C=O) in γ-Fe_2_O_3_@PEG@MAc (Fig. 4c)^40^. The peak at 1650 cm^−1^ elucidates the stretching vibrations of the carbonyl group (–CONH–) in γ-Fe_2_O_3_@PEG@PAMAM G_0_ (Fig. 4c)^40^. Also, the absorption bond recorded for γ-Fe_2_O_3_@PEG@PAMAM G_0_-Cu around 1540 cm^−1^ is attributed to the bending mode of N–H (Fig. 4c). Additionally, the bands corresponding to the asymmetric/symmetric aliphatic stretching modes of –CH_2_ are represented in the regions 2935 and 2865 cm^‒1^ (Fig. 4b–f)^41^. The broad band between 3100 and 3700 cm^‒1^ corresponds to –NH and –OH stretching vibrations and a hydrogen bond (Fig. 4b–f)^41^. Also, the peak at 1485 cm^−1^ is ascribed to the bending of the (–CH_2_–) bonds^42,43^, whereas the peak around 1220 cm^−1^ is credited to the stretching vibrations of (C–N) bond (Fig. 4b,d)^44^. These results affirmed the successful preparation of the desired γ-Fe_2_O_3_@PEG@PAMAM G_0_-Cu complex catalyst. Also, comparison of the FT-IR spectra of γ-Fe_2_O_3_@PEG@PAMAM G_0_ and γ-Fe_2_O_3_@PEG@PAMAM G_0_-Cu, showed the red shifting (~ 7 cm^−1^) within the wavenumbers of stretching and bending modes of N–H indicating that the Cu is coordinated on the surface of the nitrogen atoms of γ-Fe_2_O_3_@PEG@PAMAM G_0_ (Fig. 4c,d). These results confirmed the successful preparation of the γ-Fe_2_O_3_@PEG@PAMAM G_0_-Cu MNPs.

The XRD patterns of the purity γ-Fe_2_O_3_ and γ-Fe_2_O_3_@PEG@PAMAM G_0_-Cu catalyst is depicted in Fig. 5. The diffractogram of γ-Fe_2_O_3_ indicated several diffraction main peaks at 15°, 23.1°, 26.9°, 30.3°, 35.8°, 43.6°, 50°, 54.8°, 57.3°, 63.2°, 71.1°, and 74.4° (2θ), corresponded respectively to the (110), (210), (211), (220), (311), (400), (421), (422), (511), (440), (620), and (533) miller indices, that confirms with cubic magnetite crystal structure according to data base peaks (JCPDS card No. 39-1346) (Fig. 5a)^45,46^. As shown in XRD pattern of the γ-Fe_2_O_3_@PEG@PAMAM G_0_-Cu, crystalline phase and peaks location of this pattern also exhibit cubic structure and displayed the similar diffraction peaks to those of the pattern pertinent γ-Fe_2_O_3_. Further, in XRD pattern of γ-Fe_2_O_3_@PEG@PAMAM G_0_-Cu, decreasing of peaks intensity in comparison to γ-Fe_2_O_3_ is clearly visible which is due to the functionalization of maghemite NPs with various groups and Cu complex formation (Fig. 5b). The crystallite size of γ-Fe_2_O_3_@PEG@PAMAM G_0_-Cu catalyst was determined by using Williamson-Hall equation: *β*·cos θ = (Kλ)/D + *η*·sin θ. According to the Williamson-Hall plot, with 0% strain the particle size was found 11.4 nm (114 Å) (ESI, Fig. S1).

Scanning electron microscopy (SEM) investigation was also performed to study the surface and cross sectional morphology of the corresponding the preparation various parts of Cu catalyst, including, γ-Fe_2_O_3_ (a), γ-Fe_2_O_3_@PEG (b), γ-Fe_2_O_3_@PEG@APTES (c), γ-Fe_2_O_3_@PEG@MAc (d), γ-Fe_2_O_3_@PEG@PAMAM G_0_ (e), γ-Fe_2_O_3_@PEG@PAMAM G_0_-Cu (f), with scale bar 200 nm (Fig. 6a–f). The results of all SEM micrographs of the preparation various parts of γ-Fe_2_O_3_@PEG@PAMAM G_0_-Cu complex, clearly reveal the homogeneous spherical morphology and uniform size distribution (Fig. 6a–f). Also, the morphology of the γ-Fe_2_O_3_@PEG@PAMAM G_0_-Cu complex were monitored by transmission electron microscopy (TEM) (Fig. 5a). TEM picture clearly shows the small sized spherical structure without significant agglomeration phenomena for the γ-Fe_2_O_3_@PEG@PAMAM G_0_-Cu MNPs (Fig. 7a). Also, the average particle size of the adsorbed copper catalyst was 12 nm based on the TEM images (ESI, Fig. S2).

The EDX analysis results confirmed that γ-Fe_2_O_3_@PEG@PAMAM G_0_-Cu complex encompass the Cu, N, C, O, Si, and Fe elements, as expected (Fig. 8). In additional, EDS elemental mapping of γ-Fe_2_O_3_@PEG@PAMAM G_0_-Cu, confirmed presence all expected elements including Fe, N, C, Si, Cu, O, and shows that Cu is homogeneously distributed on the γ-Fe_2_O_3_@PEG@PAMAM G_0_-Cu (Fig. 9).

The loading of the Cu amount on catalyst was determined by ICP-OES analysis which according to the results amount of copper incorporated on 1.0 g of γ-Fe_2_O_3_@PEG@PAMAM G_0_-Cu complex was determined to be 0.96 mmol.

The TGA curve of γ-Fe_2_O_3_@PEG@PAMAM G_0_-Cu showed weight loss at five stages, totaling 18.57% that demonstrate the good thermal stability of γ-Fe_2_O_3_@PEG@PAMAM G_0_-Cu, at a heating rate of 10 °C min^–1^ in the temperature range of 25–800 °C under nitrogen atmosphere (Fig. 10).

The first weight loss is attributed to the removal of trapped water content in the crystalline structure of the Cu complex (− 1.60 W%, ~ 150 °C) (Fig. 10). The second to fifth weight loss of γ-Fe_2_O_3_@PEG@PAMAM G_0_-Cu were observed in the regions 243–600 °C (− 1.60, − 5.60, − 1.21, and − 1.64 W%) which are attributed to decomposition of the organic compounds grafted on the surface of γ-Fe_2_O_3_, including, PEG, amino-siloxy linker, and PAMAM dendrimer (Fig. 10). The weight loss, in the temperature range of 800 °C (− 7.10 W%) probably are related to the removal of PEG and N_2_, CO_2_ gases, and as well as mineral impurities in the γ-Fe_2_O_3_ (Fig. 10).

Also, for better comprehension of the thermal behavior of γ-Fe_2_O_3_@PEG@PAMAM G_0_-Cu, the thermogravimetric analysis results were compared for γ-Fe_2_O_3_, γ-Fe_2_O_3_@PEG and γ-Fe_2_O_3_@PEG@PAMAM G_0_-Cu compounds (Fig. 11). Based on the results of thermogram curves depicted in Fig. 11, of the total weight loss for the γ-Fe_2_O_3_@PEG@PAMAM G_0_-Cu (− 18.57 w%) in the temperature range of 25–800 °C, ~ − 3.99 W%, is attributed to the mineral impurities in the γ-Fe_2_O_3_ MNPs, and ~ − 4.92 W% is attributed to decomposition of PEG (Fig. 11).

The magnetic properties of γ-Fe_2_O_3_ MNPs and γ-Fe_2_O_3_@PEG@PAMAM G_0_-Cu complex were evaluated using the vibrating sample magnetometer analysis (VSM) at 25 °C (Fig. 12). According to the VSM results, the magnetization for γ-Fe_2_O_3_ MNPs was found around 55 emu g^−1^, which revealed the superparamagnetic property (Fig. 12a). After functionalization of γ-Fe_2_O_3_ to prepare γ-Fe_2_O_3_@PEG@PAMAM G_0_-Cu, this value dropped to 27 emu g^−1^ (Fig. 12b) thus confirming the successful functionalization of the γ-Fe_2_O_3_ MNPs and the formation of Cu complex (Fig. 12).

### Evaluation of the catalytic activity of γ-Fe_2_O_3_@PEG@PAMAM G_0_-Cu MNPs

**Figure 13:**
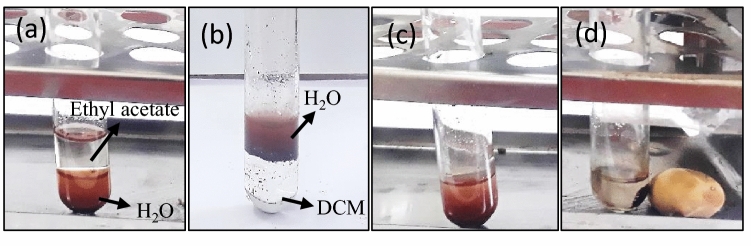
(**a**) Distribution of γ-Fe_2_O_3_@PEG@PAMAM G_0_-Cu in biphasic H_2_O-ethyl acetate, (**b**) distribution of γ-Fe_2_O_3_@PEG@PAMAM G_0_-Cu in biphasic H_2_O-dichloromethane (DCM), (**c**) dispersion of γ-Fe_2_O_3_@PEG@PAMAM G_0_-Cu in H_2_O, (**d**) easy separation of γ-Fe_2_O_3_@PEG@PAMAM G_0_-Cu using an external magnet.

Subsequent to the characterization and structural confirmation of the γ-Fe_2_O_3_@PEG@PAMAM G_0_-Cu, the Sonogashira C–C cross coupling reaction was explored for assessing its performance and reactivity in aqueous medium as a green solvent, the main objective of our strategy being to provide an active catalyst in water as an ideal alternative to conventional organic solvents for performing such coupling reactions. Figure 13 clearly show the hydrophilic properties of γ-Fe_2_O_3_@PEG@ PAMAM G_0_-Cu catalyst.

### Optimization study for the Sonogashira cross-coupling reaction

In this section, the optimal reaction conditions were explored for the Sonogashira cross-coupling reaction, using γ-Fe_2_O_3_@PEG@PAMAM G_0_-Cu as a hydrophilic catalyst. To this end, phenylacetylene and aryl iodide were selected as model substrates to identify appropriate parameters on the progress of the target reaction. Thus, the effect of temperature changes, amount of catalyst loading, type and amount of base used, on the model reaction was discerned (Table 1, entries 1–10). Initially, 0.8 mol% of Cu complex was exposed to model substrates and 2 mmol of various base (K_2_CO_3_, Et_3_N, NaOH, KOH) in water as a green solvent (5.0 mL), in pure water, at 80 °C (Table 1, entries 1–4). When the reaction was performed using K_2_CO_3_, Et_3_N bases (Table 1, entries 1 and 2), formation of 1,2-diphenylacetylene product **1a** reached to 50 and 68% yields, respectively. Also, changing the type of base to KOH resulted in an improved yield of **1a** (Table 1, 86%, entry 4). Best result was obtained for the formation of **1a**, deploying NaOH (94%, entry 3) as a base. Subsequently, the effect of temperature changes was examined on advancing the model reaction (Table 1, entries 5 and 6). In this series of experiments, the model reaction could be performed at the room temperature, even after 12 h of continuous reaction, no product was obtained (Table 1, trace, entry 5). Also, decreasing the temperature to 60 °C led to incomplete production (Table 1, 80%, entry 6). Best result was obtained at 80 °C that led to 94% formation of **1a** (Table 1, entry 3). In the next stage, amount loading of Cu catalyst was assessed where the ideal result was found to relate to 0.8 mol% loading of the catalyst (0.8 mol% of Cu, 0.006 g of Cu catalyst) that led to formation of biphenyl product **1a** in 94% yield (Table 1, entry 3). Furthermore, in the absence of NaOH as a base, no product was obtained (Table 1, entry 10). Decreasing the amount of base used (NaOH, 1 mol) also did not have a favorable effect, which afforded moderate conversion of **1a** (70%, entry 8). Also, increase the amount of base used (NaOH, 3 mol) also was not effective in progressing the model reaction (Table 1, 94%, entry 9). In general, the best results were found using 0.8 mol% loading of the γ-Fe_2_O_3_@PEG@PAMAM G_0_-Cu catalyst (0.006 g), NaOH as a base (2 mmol), in H_2_O (5 mL) at 80 °C for 180 min giving compound **1a** in 94% isolated yield (see Table 1 for more details).

**Table 1:** Optimization reaction condition of Sonogashira coupling of iodobenzene and phenylacetylene catalyzed by γ-Fe_2_O_3_@PEG@PAMAM G_0_-Cu in the water under different conditions.

Entry	Catalyst (mol%)	Base (mmol)	T (^o^C)	Time (h)	Isolated yield (%)^a^
1	0.8	K_2_CO_3_ (2)	80	3	50
2	0.8	Et_3_N (2)	80	3	68
3	0.8	NaOH (2)	80	3	94
4	0.8	KOH (2)	80	3	86
5	0.8	NaOH (2)	r.t	12	Trace
6	0.8	NaOH (2)	60	3	80
7	0.4	NaOH (2)	80	3	65
8	0.8	NaOH (1)	80	3	70
9	0.8	NaOH (3)	80	3	94
10	0.8	–	80	3	–

^a^Reaction conditions: iodobenzene (1.00 mmol), phenylacetylene (1.5 mmol), H_2_O (5.0 mL).

### Substrate scope

To the overall performance appraisement and to ascertain the limitation of Sonogashira reaction using γ-Fe_2_O_3_@PEG@PAMAM G_0_-Cu catalyst, a series of assorted aryl iodides and aryl bromides were examined in reaction with phenylacetylene under the optimal reaction conditions (0.8 mol% of Cu catalyst with NaOH, in pure water, at 80 °C, see Table 2). As depicted in the Table 2, a broad range of 1,2-diphenylacetylene products were synthesized, emanating from both, the aryl bromide and aryl iodide substituted with electron-donating (–NH_2_, –OMe, –Me) and electron-withdrawing functional groups (Cl, –CO_2_Me, –COMe, –CN, –NO_2_) and affording good to excellent yields (45–90%). However, the results related to aryl iodides (55–94%) were better than aryl bromide (45–80%), I > Br. In general, the results of using γ-Fe_2_O_3_@PEG@PAMAM G_0_-Cu for Sonogashira cross coupling reactions to the corresponding 1,2-diphenylacetylene products, were encouraging which led to the production a range of 1,2-diphenylacetylene products (see Table 2 entries 1a–p for additional details).

**Table 2:** Sonogashira coupling reactions of different aryl halides with phenylacetylene catalyzed by γ-Fe_2_O_3_@PEG@PAMAM G_0_-Cu^a^.


		
**1a**: *Hal*: I, 94%, 3 h, TON: 117.5 **1a**: *Hal*: Br, 80%, 5 h, TON: 100	**1b**: *Hal*: I, 88%, 5 h, TON: 110 **1b**: *Hal*: Br, 75%, 6 h, TON: 93	**1c**: *Hal*: I, 88%, 4 h, TON: 1110 **1c**: *Hal*: Br, 68%, 5.5 h, TON: 85
		
**1d**: *Hal*: I, 80%, 5 h, TON: 100 **1d**: *Hal*: Br, 50%, 5 h, TON: 62	**1e**: *Hal*: I, 90%, 3 h, TON: 112 **1e**: *Hal*: Br, 78%, 6 h, TON: 97	**1f.**: *Hal*: I, 80%, 4 h, TON: 100 **1f.**: *Hal*: Br, 50%, 5.5 h, TON: 62
		
**1 g**: *Hal*: I, 80%, 5 h, TON: 100 **1 g**: *Hal*: Br, 60%, 8 h, TON: 75	**1 h**: *Hal*: I, 92%, 5 h, TON: 115 **1 h**: *Hal*: Br, 80%, 5 h, TON: 100	**1i**: *Hal*: I, 90%, 6 h, TON: 112 **1i**: *Hal*: Br, 73%, 4 h, TON: 91
		
**1j**: *Hal*: I, 93%, 5 h, TON: 116 **1j**: *Hal*: Br, 75%, 7 h, TON: 93	**1 k**: *Hal*: I, 78%, 4.5 h, TON: 97 **1 k**: *Hal*: Br, 65%, 5 h, TON: 81	**1 l**: *Hal*: I, 70%, 5 h, TON: 87 **1 l**: *Hal*: Br, 54%, 6 h, TON: 72
		
**1 m**: *Hal*: I, 55%, 6 h, TON: 68 **1 m**: *Hal*: Br, 45%, 5 h, TON: 56	**1n**: *Hal*: I, 65%, 4 h, TON: 81 **1n**: *Hal*: Br, 45%, 4 h, TON: 56	**1o**: *Hal*: I, 92%, 5 h, TON: 115 **1o**: *Hal*: Br, 88%, 6 h, TON: 110
		
**1p**: *Hal*: I, 91%, 4 h, TON: 113 **1p**: *Hal*: Br, 85%, 6 h, TON: 106		

^a^Reaction conditions: aryl halide (1.00 mmol), acetylene (1.5 mmol), NaOH (2.00) mmol, catalyst (0.006 g, 0.8 mol% Cu), H_2_O (5.0 mL), 80 °C; All yields are isolated.

### Experimental controls

**Figure 14:**
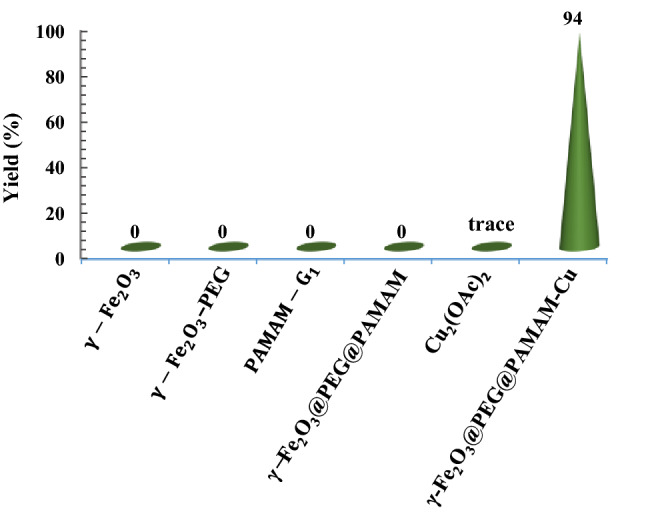
Control experiments to show the catalytic activity of the γ-Fe_2_O_3_@PEG@PAMAM G_0_-Cu for the Sonogashira model reaction.

To demonstrate the catalytic role and uniqueness of the nano γ-Fe_2_O_3_@PEG@PAMAM G_0_-Cu complex, some control experiments were conducted for the model reaction of Sonogashira (Fig. 14). For this goal, the model reaction was performed and compared using the parent materials. The results clearly indicated the high activity and efficiency of the γ-Fe_2_O_3_@PEG@PAMAM G_0_-Cu catalyst, compared to the other examined catalysts (Fig. 14).

### Recyclability study

Recyclability aspects of the γ-Fe_2_O_3_@PEG@PAMAM G_0_-Cu catalyst complex were evaluated for model reaction to achieve of **1a**, under the optimized reaction conditions for 6 serial runs (see Table 3). According to the obtained results, the highly activated catalyst represented just 0–1% loss of efficiency after the 6 successive runs for preparation of **1a** (Table 3).

**Table 3:** Recyclability of the γ-Fe_2_O_3_@PEG@PAMAM G_0_-Cu catalyst in performed of the Sonogashira reaction^a^.

Run^a^	Yield (%)	Time (h)	TON
1	94	3	177
2	94	3	177
3	94	4	177
4	93	4	116
5	93	4	116
6	93	5	116

^a^Reaction conditions: iodobenzene (1.00 mmol), phenylacetylene (1.5 mmol), NaOH (2.00 mmol), catalyst (0.8 mol% of Cu, 0.006 g), H_2_O (5.0 mL), 80 °C.

**Figure 15:**
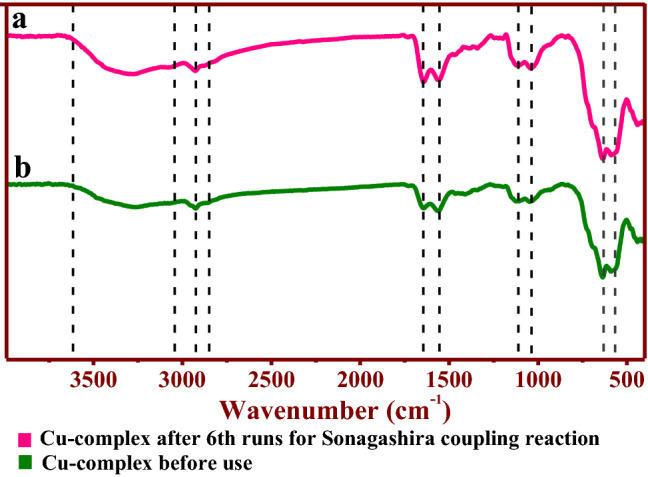
FT‒IR spectra of (**a**) γ-Fe_2_O_3_@PEG@PAMAM-Cu, before use; (**b**) γ-Fe_2_O_3_@PEG@PAMAM G_0_-Cu catalyst after 6th runs for the model reaction.

In addition, FT–IR analysis was performed for the γ-Fe_2_O_3_@PEG@PAMAM G_0_-Cu catalyst used after the 6th runs in the model reaction (see Fig. 15a,b). Study of the FT–IR spectra related to 6th runs of catalyst, demonstrate the stability structure of the catalyst that have been retained after 6th recovery in the model reaction (see Fig. 15).

**Figure 16:**
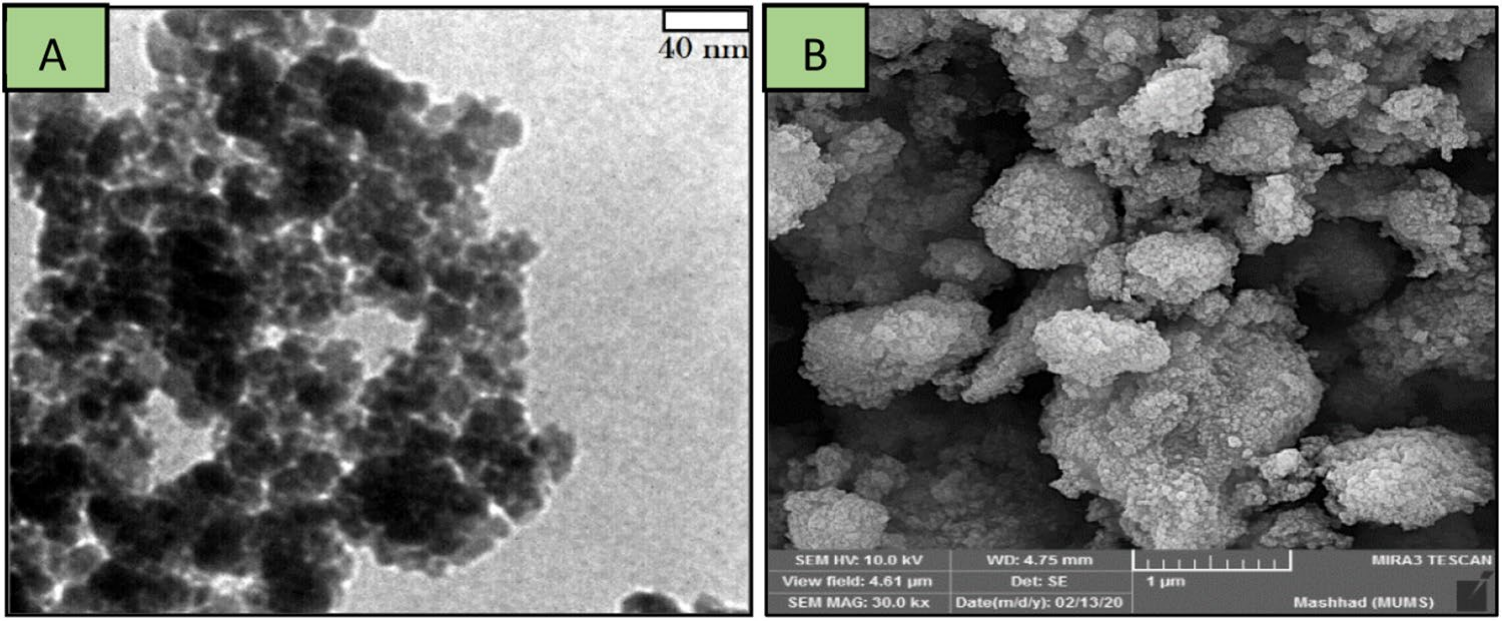
(**A**) TEM, and (**B**) FE − SEM, images recovered of the γ-Fe_2_O_3_@PEG@PAMAM G_0_-Cu complex after the 6th run for model reaction, reaction conditions: iodobenzene (1.00 mmol), phenylacetylene (1.5 mmol), NaOH (2.0 mmol), catalyst (0.8 mol% of Cu, 0.006 g), H_2_O (5.0 mL), 80 °C.

The comparison of TEM and SEM images of fresh copper catalyst and the used catalyst after 6th run in the model reaction, did not reveal any detectable and noticeable change in size and morphology (Fig. 16A, B). Moreover, a good dispersion and small NPs of Cu-complex even after six recovering times, is related to the stability of the γ-Fe_2_O_3_@PEG@PAMAM G_0_-Cu catalyst. However, minimal agglomeration can be seen in the TEM image that is associated with the dipole–dipole interactions of magnetic nano particles (Fig. 16A, B).

**Figure 17:**
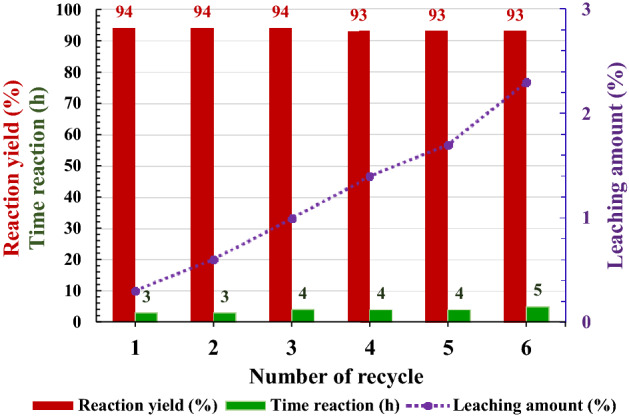
Determination, amount metal leaching of the Cu catalyst during 6th runs of the model reaction, in optimal reaction conditions; reaction conditions: iodobenzene (1.00 mmol), phenylacetylene (1.5 mmol), NaOH (2.0 mmol), catalyst (0.006 g, 0.8 mol% of Cu), H_2_O (5.0 mL), 80 °C.

ICP analysis was performed for measurement of the copper leaching for the six catalytic run steps of model reaction (Fig. 17). ICP analysis results of Cu complex after the 6th run demonstrated a stable catalytic structure with a small amounts of Cu leaching (average up to 2.4%) (Fig. 17).

Moreover, a rigorous investigation was conducted to elucidate the stability and heterogeneous nature of the catalyst using the hot-filtration method on the model reaction (ESI, Fig. S3). After the catalyst was magnetically removed from the reaction medium (t = 70 min, 46% yield of 1,2-diphenylacetylene monitored by GC), and the reactants were allowed to undergo further reaction, when the reaction did not progress (49% yield after 180 min, Fig. S3). This confirmed that the catalyst functioned heterogeneously in the reaction and consequently only slight leaching occurred during the reaction.

### Catalytic performance

Catalytic efficiency of the γ-Fe_2_O_3_@PEG@PAMAM G_0_-Cu complex in terms of the reaction time, and yield was compared with the other reported catalysts for the Sonogashira reaction (see Table 4). The high activity of the catalyst in water media, as an abundant, and available solvent is notable. Additional benefits of this catalytic methodology in comparison with the others catalytic protocols are presented in Table 4 are the simplicity of catalyst separation, higher yield of the corresponding products during the shorter reaction times, eco-friendly and economic aspects, as well as the use of available and inexpensive copper metal complex (see Table 4. For more details).

**Table 4:** Catalytic activity of the γ-Fe_2_O_3_@PEG@PAMAM G_0_-Cu MNPs was compared with reported catalysts for the Sonogashira cross coupling reaction (1a)^a^.

Entry	Catalyst (mol%)	Reaction conditions	Time (h)	Yield^b^ (%)	Refs.
1	CuI (0.2 mol%)	K_2_CO_3_/PPh_3_ (4 mol%)/H_2_O/140 °C	24	43	^47^
2	NHC precursor (20 mol%)/CuSO_4_·5H_2_O (20 mol%)^c^	K_2_CO_3_/DMF/125 °C	8	71	^48^
3	Co(C_9_H_9_NO_2_)_3_	K_2_CO_3_/DMF/ethylene glycol/r.t	8	82	^49^
4	MNPs@Cs-MS-Co (0.55 mol%)^*d*^	KOH/DMSO/140 °C	10	72	^50^
5	CuO/TiO_2_	K_2_CO_3_/EtOH/r.t	15	54	^51^
6	Pd_3_Cu_1_/SiC	Cs_2_CO_3_/DMF/60 °C	8	99	^52^
7	Cu(OAc)_2_ (10 mol%)/*β*-diketone	K_2_CO_3_/DMF/120 °C	36	90	^53^
8	Cu-Salen (10 mol%)/PTC^*e*^	NaOH/H_2_O/100 °C	24	76	^54^
9	Cu (10 mol%)/DABCO@SiO	KOH/DMF/140 °C	2	94	^55^
10	γ-Fe_2_O_3_@PEG@PAMAM G_0_-Cu	NaOH/H_2_O/80 °C	3	94	This work

^a^Sonogashira: reaction of iodobenzene and phenyl acetylene.

^b^Isolated yield.

^c^NHC: N-heterocyclic carbine.

^d^MNPs@Cs-MS-Co: cobalt tagged on MNPs-chitosan functionalized with methyl salicylate.

^e^PTC: phase-transfer catalyst.

## Summary and conclusions

In brief, we have developed an environmentally friendly and economical methodology using a water-dispersible, recyclable copper catalyst (γ-Fe_2_O_3_@PEG@PAMAM G_0_-Cu) based on the γ-Fe_2_O_3_ nanoparticles coated with polyethylene glycol to promote the Sonogashira coupling reaction in water as a solvent. The physicochemical properties of γ-Fe_2_O_3_@PEG@PAMAM G_0_-Cu catalyst were characterized using various techniques such as, FT-IR, FE-SEM, XRD, VSM, EDS, TGA and TEM analysis. Polyethylene glycol on the surface of iron oxide nanoparticles instigated the good dispersion of the catalyst in the aqueous media and thus increased the interaction between the precursor materials and the catalyst like homogeneous systems. A wide range of aryl halides (aryl iodide and aryl bromide) reacted with phenylacetylene using copper catalyst in the water and led to attainment of the desired products with good to excellent efficiencies. The easy recyclability of the catalyst using an external magnet and its reusability, as well as the good performance of the catalyst in water solvent without the use of additives such as alcohol or added surfactant are other salient advantages of this developed catalyst.

## Supplementary Information

Supplementary Information.

## Data Availability

All data generated or analyzed during this study are included in this published article [and its supplementary information files].
