# Supplementary material for: Copper adorned magnetic nanoparticles as a heterogeneous catalyst for Sonogashira coupling reaction in aqueous media
Source: Sci Rep. 2022 Oct 26;12:17986. doi: 10.1038/s41598-022-22567-5 (PMC9606120; doi:10.1038/s41598-022-22567-5)
Supplement: Supplementary file 1 — Supplementary Information. [file 41598_2022_22567_MOESM1_ESM.docx]

**Copper Adorned Magnetic Nanoparticles as a Heterogeneous Catalyst for Sonogashira Coupling Reaction in Aqueous Media**

Safoora Sheikh ^1,2^*, Mohammad Ali Nasseri ^1^*, Ali Allahresani ^1^, and Rajender S. Varma^3^

*^1^Department of Chemistry, Faculty of Basic Sciences, University of Birjand, P. O. Box 97175-615, Birjand, Iran*

*^2^Institut für Organische Chemie, Universität Regensburg, Universitätsstr. 31, 93053 Regensburg, Germany*

*^3^Regional Centre of Advanced Technologies and Materials, Czech Advanced Technology and Research Institute, Palacký University in Olomouc, ˇSlechtitelů 27, 783 71, Olomouc, Czech Republic.*

*To whom correspondence should be addressed:

*sheikhsafoora90@gmail.com; [Safoora.sheikh@chemi.uni_regensburg.de](mailto:Safoora.sheikh@chemi.uni_regensburg.de) (S. Sheik)

[*manaseri@birjand.ac.ir](mailto:*manaseri@birjand.ac.ir); Tel: +98-561-32202065 (M. A. Nasseri)

**General**

All starting materials were commercially available and were utilized as received. Thin layer chromatography (TLC) was performed on silica–gel 60 F254 plates and UV light was used for visualization. Melting points were determined on a Tropical Labequip apparatus. The FT–IR spectra (JASCO–FT–IR 4600) were recorded using KBr pellet. The ^1^H–NMR and ^13^C–NMR spectra were recorded on a Bruker Avance DPX–300 spectrometer in the deuterated solvents (CDCl_3_), using tetramethylsilane (TMS) as an internal standard. The Field emission scanning electron microscopy (FE–SEM) images of the prepared catalysts were recorded on a Tescan MIRA3. EDX analysis were performed using a FESEM (JEOL–7600F–Oxford) equipped with a spectrometer of energy dispersion of X–ray. The presence of the elements was confirmed using the point elemental mapping (Tescan–Mira 3–SAMX). Liquid chromatography–mass spectrometry for the PAMAM dendrimer G_1_ was recorded by LC–Mass–AB SCIEX–Q–trap 3200 instrument. The microscopic images and size distribution of the catalyst NPs were performed using TEM (Philips–EM208) operating at 100 kV voltage. The X‒ray diffraction (XRD) pattern were recorded by an X’pertpro (Philips) instrument employing Cu Kα radiation (λ = 1.5418 Å), at a scanning speed of 2 °C/min from 10 to 80 °C (2θ). TGA analysis of the samples were performed using a Q600 model from TA company under nitrogen atmosphere with a heating rate of 15 °C/min in the temperature range of 25–800 °C. The content of Pd in the catalyst was determined by OPTIMA 7300DV ICP analyzer. All the measured yields refer to the isolated products after purification by column chromatography. All the products were characterized by NMR spectroscopy.

**Figure S1**. Williamson Hall plot, determination size and strain of γ-Fe_2_O_3_@PEG@PAMAM G_0_-Cu
MNPs.

**Figure S2.** (a) Particles size distribution histogram of the γ-Fe_2_O_3_@PEG@PAMAM G_0_-Cu MNPs.

**Figure S3.** : Iodobenzene (1.00 mmol), phenylacetylene (1.5 mmol), NaOH (2.0 mmol), catalyst (0.006 g, 0.8 mol% of Cu), H_2_O (5.0 mL), 80 ^o^C; Full conversion (blue squares) is reached after 180 min. In a second run (red triangles), the catalyst was removed by filtration after 60 min, upon which no further conversion was observed.

**Spectra data for the Sonogashira cross-coupling products:**

**1,2-diphenylacetylene** (**1a**). This compound is known in the literature [[1](#_ENREF_1)].

Phenylacetylene (153.1 mg, 1.5 mmol) was reacted with iodobenzene (204.0 mg, 1.00 mmol), NaOH (80.0 mg, 2.00 mmol) and γ-Fe_2_O_3_@PEG@PAMAM G_0_-Cu (0.8 mol%, 0.006 g) in water (5 mL) at 80 °C to yield 94% (175.9 mg) 1,2-diphenylacetylene (**1a**). Chromatography: *n*-hexane/EtOAc, 8:2. **^1^H-NMR** (250 MHz, CDCl_3_) *δ* 7.23-7.47 (m, 10H); **^13^C-NMR** (62.9 MHz, CDCl_3_) *δ* 89.5, 123.3, 128.3, 129.2, 131.6.

Phenylacetylene (153.1 mg, 1.5 mmol) was reacted with bromobenzene (157.0 mg, 1.00 mmol), NaOH (80.0 mg, 2.00 mmol) and γ-Fe_2_O_3_@PEG@PAMAM G_0_-Cu (0.8 mol%, 0.006 g) in water (5 mL) at 80 °C to yield 80% (149.7 mg) 1,2-diphenylacetylene (**1a**). Chromatography: *n*-hexane/EtOAc, 8:2. **^1^H-NMR** (250 MHz, CDCl_3_) *δ* 7.23-7.45 (m, 10H); **^13^C-NMR** (62.9 MHz, CDCl_3_) *δ* 89.5, 123.4, 128.1, 129.6, 131.7.

**4-(phenylethynyl)aniline** (**1b**). This compound is well known in the literature [[1](#_ENREF_1)].

Phenylacetylene (153.1 mg, 1.5 mmol) was reacted with 4-iodoaniline (219.0 mg, 1.00 mmol), NaOH (80.0 mg, 2.00 mmol) and γ-Fe_2_O_3_@PEG@PAMAM G_0_-Cu (0.8 mol%, 0.006 g) in water (5 mL) at 80 °C to yield 88% (122.3 mg) 4-(phenylethynyl)aniline (**1b**). Chromatography: *n*-hexane/EtOAc, 8:2. **^1^H-NMR** (250 MHz, CDCl_3_) *δ* 3.62 (s, 2H, NH_2_), 6.52-7.40 (m, 9H, Ar-H); **^13^C-NMR** (62.9 MHz, CDCl_3_) *δ* 87.3, 90.2, 112.5, 114.7, 123.9, 127.7, 128.3, 131.3, 132.9, 146.7.

Phenylacetylene (153.1 mg, 1.5 mmol) was reacted with 4-bromoaniline (172.0 mg, 1.00 mmol), NaOH (80.0 mg, 2.00 mmol) and γ-Fe_2_O_3_@PEG@PAMAM G_0_-Cu (0.8 mol%, 0.006 g) in water (5 mL) at 80 °C to yield 75% (144.7 mg) 4-(phenylethynyl)aniline (**1b**). Chromatography: *n*-hexane/EtOAc, 8:2. **^1^H-NMR** (250 MHz, CDCl_3_) *δ* 3.62 (s, 2H, NH_2_), 6.53-7.42 (m, 9H, Ar-H); **^13^C-NMR** (62.9 MHz, CDCl_3_) *δ* 87.5, 90.2, 112.3, 114.8, 123.7, 127.7, 128.1, 131.3, 132.9, 146.4.

**1-methoxy-4-(phenylethynyl)benzene** (**1c**). This compound is known in the literature [[2](#_ENREF_2)].

Phenylacetylene (153.1 mg, 1.5 mmol) was reacted with 1-iodo-4-methoxybenzene (234.0 mg, 1.00 mmol), NaOH (80.0 mg, 2.00 mmol) and γ-Fe_2_O_3_@PEG@PAMAM G_0_-Cu (0.8 mol%, 0.006 g) in water (5 mL) at 80 °C to yield 83% (172.6 mg) 1-methoxy-4-(phenylethynyl)benzene (**1c**). Chromatography: *n*-hexane/EtOAc, 8:2. **^1^H-NMR** (250 MHz, CDCl_3_) *δ* 3.76 (s, 3H, CH_3_), 6.79-7.44 (m, 9H, Ar-H); **^13^C-NMR** (62.9 MHz, CDCl_3_) *δ* 55.2, 88.0, 89.4, 114.0, 115.3, 123.6, 127.9, 128.3, 131.4, 133.0, 159.6.

Phenylacetylene (153.1 mg, 1.5 mmol) was reacted with 1-bromo-4-methoxybenzene (187.0 mg, 1.00 mmol), NaOH (80.0 mg, 2.00 mmol) and γ-Fe_2_O_3_@PEG@PAMAM G_0_-Cu (0.8 mol%, 0.006 g) in water (5 mL) at 80 °C to yield 68% (141.4 mg) 1-methoxy-4-(phenylethynyl)benzene (**1c**). Chromatography: *n*-hexane/EtOAc, 8:2. **^1^H-NMR** (250 MHz, CDCl_3_) *δ* 3.72 (s, 3H, CH_3_), 6.75-7.44 (m, 9H, Ar-H); **^13^C-NMR** (62.9 MHz, CDCl_3_) *δ* 55.2, 88.1, 89.3, 114.3, 115.4, 123.5, 127.9, 128.7, 131.5, 133.3, 159.8.

**1-methoxy-2-(phenylethynyl)benzene** (**1d**). This compound is known in the literature [[1](#_ENREF_1)].

Phenylacetylene (153.1 mg, 1.5 mmol) was reacted with 2-iodoanisole (243.0 mg, 1.00 mmol), NaOH (80.0 mg, 2.00 mmol) and γ-Fe_2_O_3_@PEG@PAMAM G_0_-Cu (0.8 mol%, 0.006 g) in water (5 mL) at 80 °C to yield 80% (166.5 mg) 1-methoxy-2-(phenylethynyl)benzene (**1d**). Chromatography: *n*-hexane/EtOAc, 8:2. **^1^H NMR** (250 MHz, CDCl_3_) *δ* 3.82 (s, 3H, CH_3_), 6.87-7.53 (m, 9H, Ar-H); **^13^C NMR** (CDCl_3_, 62.9 MHz) *δ* 55.8, 85.6, 93.3, 110.6, 112.4, 120.4, 123.5, 128.0, 128.1, 129.7, 131.6, 133.5, 159.8.

Phenylacetylene (153.1 mg, 1.5 mmol) was reacted with 2-bromoanisole (187.0 mg, 1.00 mmol), NaOH (80.0 mg, 2.00 mmol) and γ-Fe_2_O_3_@PEG@PAMAM G_0_-Cu (0.8 mol%, 0.006 g) in water (5 mL) at 80 °C to yield 50% (104.1 mg) 1-methoxy-2-(phenylethynyl)benzene (**1d**). Chromatography: *n*-hexane/EtOAc, 8:2. **^1^H NMR** (250 MHz, CDCl_3_) *δ* 3.83 (s, 3H, CH_3_), 6.89-7.56 (m, 9H, Ar-H); **^13^C NMR** (CDCl_3_, 62.9 MHz) *δ* 55.9, 85.4, 93.0, 110.4, 112.6, 120.3, 123.2, 128.0, 128.4, 129.7, 131.8, 133.6, 159.7.

**1-methyl-4-(phenylethynyl)benzene** (**1e**). This compound is known in the literature [[1](#_ENREF_1)].

Phenylacetylene (153.1 mg, 1.5 mmol) was reacted with 1-iodo-4-methylbenzene (218.0 mg, 1.00 mmol), NaOH (80.0 mg, 2.00 mmol) and γ-Fe_2_O_3_@PEG@PAMAM G_0_-Cu (0.8 mol%, 0.006 g) in water (5 mL) at 80 °C to yield 90% (172.8 mg) 1-methyl-4-(phenylethynyl)benzene (**1e**). Chromatography: *n*-hexane/EtOAc, 8:2. **^1^H-NMR** (CDCl_3_, 250 MHz) *δ* 2.22 (s, 3H, CH_3_), 7.14-7.42 (m, 9H, Ar-H); **^13^C-NMR** (62.9 MHz, CDCl_3_) *δ* 21.5, 88.7, 89.6, 120.2, 123.5, 128.1, 128.3, 129.1, 131.5, 131.7, 138.4.

Phenylacetylene (153.1 mg, 1.5 mmol) was reacted with 1-bromo-4-methylbenzene (171.0 mg, 1.00 mmol), NaOH (80.0 mg, 2.00 mmol) and γ-Fe_2_O_3_@PEG@PAMAM G_0_-Cu (0.8 mol%, 0.006 g) in water (5 mL) at 80 °C to yield 78% (149.7 mg) 1-methyl-4-(phenylethynyl)benzene (**1e**). Chromatography: *n*-hexane/EtOAc, 8:2. **^1^H-NMR** (CDCl_3_, 250 MHz) *δ* 2.24 (s, 3H, CH_3_), 7.15-7.41 (m, 9H, Ar-H); **^13^C-NMR** (62.9 MHz, CDCl_3_) *δ* 21.8, 88.9, 89.5, 120.1, 123.7, 128.2, 128.3, 129.1, 131.6, 131.8, 138.4.

**1-methyl-2-(phenylethynyl)benzene** (**1f**). This compound is known in the literature [[1](#_ENREF_1)].

Phenylacetylene (153.1 mg, 1.5 mmol) was reacted with 1-iodi-2-methylbenzene (218.0 mg, 1.00 mmol), NaOH (80.0 mg, 2.00 mmol) and γ-Fe_2_O_3_@PEG@PAMAM G_0_-Cu (0.8 mol%, 0.006 g) in water (5 mL) at 80 °C to yield 80% (153.6 mg) 1-methyl-2-(phenylethynyl)benzene (**1m**). Chromatography: *n*-hexane/EtOAc, 8:2. **^1^H NMR** (250 MHz, CDCl_3_) *δ* 2.35 (s, 3H, CH_3_), 7.14-7.45 (m, 9H, Ar-H); **^13^C NMR** (CDCl_3_, 62.9 MHz) *δ* 21.5, 88.5, 120.3, 123.6, 128.2, 128.3, 129.4, 131.3, 131.7, 138.7.

Phenylacetylene (153.1 mg, 1.5 mmol) was reacted with 1-bromo-2-methylbenzene (171.0 mg, 1.00 mmol), NaOH (80.0 mg, 2.00 mmol) and γ-Fe_2_O_3_@PEG@PAMAM G_0_-Cu (0.8 mol%, 0.006 g) in water (5 mL) at 80 °C to yield 50% (96.0 mg) 1-methyl-2-(phenylethynyl)benzene (**1f**). Chromatography: *n*-hexane/EtOAc, 8:2. **^1^H NMR** (250 MHz, CDCl_3_) *δ* 2.35 (s, 3H, CH_3_), 7.16-7.45 (m, 9H, Ar-H); **^13^C NMR** (CDCl_3_, 62.9 MHz) *δ* 21.5, 88.6, 120.3, 123.6, 128.1, 128.3, 129.2, 131.4, 131.7, 138.5.

**1-chloro-4-(phenylethynyl)benzene** (**1g**). This compound is known in the literature [[1](#_ENREF_1)].

Phenylacetylene (153.1 mg, 1.5 mmol) was reacted with 1-chloro-4-iodobenzene (238.4 mg, 1.00 mmol), NaOH (80.0 mg, 2.00 mmol) and γ-Fe_2_O_3_@PEG@PAMAM G_0_-Cu (0.8 mol%, 0.006 g) in water (5 mL) at 80 °C to yield 80% (170.0 mg) 1-chloro-4-(phenylethynyl)benzene (**1g**). Chromatography: *n*-hexane/EtOAc, 8:2. **^1^H NMR** (250 MHz, CDCl_3_) *δ* 7.13-7.43 (m, 9H, Ar-H) ppm; **^13^C NMR** (62.9 MHz, CDCl_3_) *δ* 88.2, 90.3, 121.8, 122.9, 128.4, 128.5, 128.7, 131.6, 132.8, 134.2.

Phenylacetylene (153.1 mg, 1.5 mmol) was reacted with 1-brom-4-iod-benzol (282.9 mg, 1.00 mmol), NaOH (80.0 mg, 2.00 mmol) and γ-Fe_2_O_3_@PEG@PAMAM G_0_-Cu (0.8 mol%, 0.006 g) in water (5 mL) at 80 °C to yield 60% (127.5 mg) 1-chloro-4-(phenylethynyl)benzene (**1g**). Chromatography: *n*-hexane/EtOAc, 8:2. **^1^H NMR** (250 MHz, CDCl_3_) *δ* 7.14-7.42 (m, 9H, Ar-H); **^13^C NMR** (62.9 MHz, CDCl_3_) *δ* 88.1, 90.5, 121.9, 122.9, 128.3, 128.5, 128.8, 131.5, 132.8, 134.3.

**4-(phenylethynyl)benzaldehyde** (**1h**). This compound is known in the literature [[3](#_ENREF_3)].

Phenylacetylene (153.1 mg, 1.5 mmol) was reacted with 4-iodobenzaldehyde (232.0 mg, 1.00 mmol), NaOH (80.0 mg, 2.00 mmol) and γ-Fe_2_O_3_@PEG@PAMAM G_0_-Cu (0.8 mol%, 0.006 g) in water (5 mL) at 80 °C to yield 92% (189.7 mg) 4-(phenylethynyl)benzaldehyde (**1h**). Chromatography: *n*-hexane/EtOAc, 8:2. **^1^H-NMR** (CDCl_3_, 250 MHz) *δ* 7.36-7.87 (m, 9H, Ar-H), 10.01 (s, 1H); **^13^C-NMR** (62.9 MHz, CDCl_3_) *δ* 88.4, 93.3, 122.4, 128.4, 128.8, 129.5, 131.7, 132.0, 153.2.

Phenylacetylene (153.1 mg, 1.5 mmol) was reacted with 4-bromobenzaldehyde (185.0 mg, 1.00 mmol), NaOH (80.0 mg, 2.00 mmol) and γ-Fe_2_O_3_@PEG@PAMAM G_0_-Cu (0.8 mol%, 0.006 g) in water (5 mL) at 80 °C to yield 80% (164.9 mg) 4-(phenylethynyl)benzaldehyde (**1h**). Chromatography: *n*-hexane/EtOAc, 8:2. **^1^H-NMR** (CDCl_3_, 250 MHz) *δ* 7.36-7.87 (m, 9H, Ar-H), 10.03 (s, 1H); **^13^C-NMR** (62.9 MHz, CDCl_3_) *δ* 88.4, 93.1, 122.3, 128.2, 128.4, 129.5, 131.5, 132.6, 153.1.

**methyl 4-(phenylethynyl)benzoate** (**1i**). This compound is known in the literature [[3](#_ENREF_3)].

Phenylacetylene (153.1 mg, 1.5 mmol) was reacted with methyl-4-iodobenzoat (262.0 mg, 1.00 mmol), NaOH (80.0 mg, 2.00 mmol) and γ-Fe_2_O_3_@PEG@PAMAM G_0_-Cu (0.8 mol%, 0.006 g) in water (5 mL) at 80 °C to yield 90% (212.5 mg) methyl 4-(phenylethynyl)benzoate (**1i**). Chromatography: *n*-hexane/EtOAc, 8:2. **^1^H-NMR** (CDCl_3_, 250 MHz) *δ* 3.39 (s, 3H), 7.36-8.08 (m, 9H, Ar-H); **^13^C-NMR** (62.9 MHz, CDCl_3_) *δ* 53.2, 88.8, 92.5, 122.8, 128.2, 128.9, 129.7, 131.5, 131.9, 166.5.

Phenylacetylene (153.1 mg, 1.5 mmol) was reacted with methyl-4-bromobenzoate (215.0 mg, 1.00 mmol), NaOH (80.0 mg, 2.00 mmol) and γ-Fe_2_O_3_@PEG@PAMAM G_0_-Cu (0.8 mol%, 0.006 g) in water (5 mL) at 80 °C to yield 73% (172.4 mg) methyl 4-(phenylethynyl)benzoate (**1i**). Chromatography: *n*-hexane/EtOAc, 8:2. **^1^H-NMR** (CDCl_3_, 250 MHz) *δ* 3.39 (s, 3H), 7.35-8.07 (m, 9H, Ar-H); **^13^C-NMR** (62.9 MHz, CDCl_3_) *δ* 53.1, 88.6, 92.4, 122.8, 128.4, 128.9, 129.6, 131.5, 131.8, 166.5.

**1-(4-(phenylethynyl)phenyl)ethanone** (**1j**). This compound is known in the literature [[1](#_ENREF_1)].

Phenylacetylene (153.1 mg, 1.5 mmol) was reacted with 1-(4-iodophenyl)ethanone (246.0 mg, 1.00 mmol), NaOH (80.0 mg, 2.00 mmol) and γ-Fe_2_O_3_@PEG@PAMAM G_0_-Cu (0.8 mol%, 0.006 g) in water (5 mL) at 80 °C to yield 93% (204.7 mg) 1-(4-(phenylethynyl)phenyl)ethanone (**1j**). Chromatography: *n*-hexane/EtOAc, 8:2. **^1^H-NMR** (250 MHz, CDCl_3_) *δ* 2.51 (s, 3H, CH_3_), 7.16- 7.86 (m, 9H, Ar-H); **^13^C-NMR** (62.9 MHz, CDCl_3_) *δ* 27.0, 88.9, 92.9, 123.0, 128.2, 128.3, 128.8, 129.5, 132.0, 132.2, 136.5, 197.6.

Phenylacetylene (153.1 mg, 1.5 mmol) was reacted with 1-(4-bromophenyl)ethanone (199.0 mg, 1.00 mmol), NaOH (80.0 mg, 2.00 mmol) and γ-Fe_2_O_3_@PEG@PAMAM G_0_-Cu (0.8 mol%, 0.006 g) in water (5 mL) at 80 °C to yield 75% (165.1 mg) 1-(4-(phenylethynyl)phenyl)ethanone (**1j**). Chromatography: *n*-hexane/EtOAc, 8:2. **^1^H-NMR** (250 MHz, CDCl_3_) *δ* 2.52 (s, 3H, CH_3_), 7.14- 7.85 (m, 9H, Ar-H); **^13^C-NMR** (62.9 MHz, CDCl_3_) *δ* 27.2, 88.8, 92.9, 123.3, 128.1, 128.2, 128.7, 129.6, 132.0, 132.2, 136.4, 197.5.

**4-(phenylethynyl)benzonitrile** (**1k**). This compound is known in the literature [[1](#_ENREF_1)].

Phenylacetylene (153.1 mg, 1.5 mmol) was reacted with 4-iod-benzonitril (229.0 mg, 1.00 mmol), NaOH (80.0 mg, 2.00 mmol) and γ-Fe_2_O_3_@PEG@PAMAM G_0_-Cu (0.8 mol%, 0.006 g) in water (5 mL) at 80 °C to yield 78% (158.4 mg) 4-(phenylethynyl)benzonitrile (**1k**). Chromatography: *n*-hexane/EtOAc, 8:2. **^1^H-NMR** (250 MHz, CDCl_3_) *δ* 7.28-7.51 (m, 9H, Ar-H); **^13^C-NMR** (62.9 MHz, CDCl_3_) *δ* 87.8, 93.6, 111.8, 118.4, 122.1, 128.2, 128.5, 129.3, 131.7, 132.0, 132.0.

Phenylacetylene (153.1 mg, 1.5 mmol) was reacted with 4-bromo-benzonitril (182.0 mg, 1.00 mmol), NaOH (80.0 mg, 2.00 mmol) and γ-Fe_2_O_3_@PEG@PAMAM G_0_-Cu (0.8 mol%, 0.006 g) in water (5 mL) at 80 °C to yield 65% (132.0 mg) 4-(phenylethynyl)benzonitrile (**1k**). Chromatography: *n*-hexane/EtOAc, 8:2. **^1^H-NMR** (250 MHz, CDCl_3_) *δ* 7.28-7.53 (m, 9H, Ar-H); **^13^C-NMR** (62.9 MHz, CDCl_3_) *δ* 87.7, 93.7, 111.4, 118.5, 122.2, 128.2, 128.5, 129.1, 131.7, 132.03, 132.06.

**1-nitro-4-(phenylethynyl)benzene** (**1l**). This compound is known in the literature [[1](#_ENREF_1)].

Phenylacetylene (153.1 mg, 1.5 mmol) was reacted with 1-iodo-4-nitrobenzene (249.0 mg, 1.00 mmol), NaOH (80.0 mg, 2.00 mmol) and γ-Fe_2_O_3_@PEG@PAMAM G_0_-Cu (0.8 mol%, 0.006 g) in water (5 mL) at 80 °C to yield 70% (156.2 mg) 1-nitro-4-(phenylethynyl)benzene (**1l**). Chromatography: *n*-hexane/EtOAc, 8:2. **^1^H-NMR** (250 MHz, CDCl_3_) *δ* 7.37-8.22 (m, 9H, Ar-H); **^13^C-NMR** (62.9 MHz, CDCl_3_) *δ* 87.5, 94.7, 122.0, 123.6, 128.5, 129.2, 130.2, 131.8, 132.2, 146.9.

Phenylacetylene (153.1 mg, 1.5 mmol) was reacted with 1-bromo-4-nitrobenzene (202.0 mg, 1.00 mmol), NaOH (80.0 mg, 2.00 mmol) and γ-Fe_2_O_3_@PEG@PAMAM G_0_-Cu (0.8 mol%, 0.006 g) in water (5 mL) at 80 °C to yield 54% (120.5 mg) 1-nitro-4-(phenylethynyl)benzene (**1l**). Chromatography: *n*-hexane/EtOAc, 8:2. **^1^H-NMR** (250 MHz, CDCl_3_) *δ* 7.36-8.22 (m, 9H, Ar-H); **^13^C-NMR** (62.9 MHz, CDCl_3_) *δ* 87.4, 94.6, 122.2, 123.8, 128.6, 129.1, 130.1, 131.5, 132.2, 146.8.

**1-nitro-3-(phenylethynyl)benzene** (**1m**). This compound is known in the literature [[4](#_ENREF_4)].

Phenylacetylene (153.1 mg, 1.5 mmol) was reacted with 1-iodo-3-nitrobenzene (249.0 mg, 1.00 mmol), NaOH (80.0 mg, 2.00 mmol) and γ-Fe_2_O_3_@PEG@PAMAM G_0_-Cu (0.8 mol%, 0.006 g) in water (5 mL) at 80 °C to yield 55% (122.7 mg) 1-nitro-3-(phenylethynyl)benzene (**1m**). Chromatography: *n*-hexane/EtOAc, 8:2. **^1^H-NMR** (400 MHz, CDCl_3_) *δ* 7.34-8.28 (m, 9H, Ar-H); **^13^C-NMR** (62.9 MHz, CDCl_3_) *δ* 87.5, 89.8, 120.2, 121.7, 123.0, 124.2, 125.5, 126.2, 127.5, 133.1, 136.5, 149.4.

Phenylacetylene (153.1 mg, 1.5 mmol) was reacted with 1-bromo-3-nitrobenzene (202.0 mg, 1.00 mmol), NaOH (80.0 mg, 2.00 mmol) and γ-Fe_2_O_3_@PEG@PAMAM G_0_-Cu (0.8 mol%, 0.006 g) in water (5 mL) at 80 °C to yield 45% (100.4 mg) 1-nitro-3-(phenylethynyl)benzene (**1m**). Chromatography: *n*-hexane/EtOAc, 8:2. **^1^H-NMR** (400 MHz, CDCl_3_) *δ* 7.36-8.29 (m, 9H, Ar-H); **^13^C-NMR** (62.9 MHz, CDCl_3_) *δ* 87.5, 89.9, 120.-, 121.6, 123.2, 124.2, 125.8, 126.0, 127.4, 133.1, 136.5, 149.5.

**1-nitro-2-(phenylethynyl)benzene** (**1n**). This compound is known in the literature [[5](#_ENREF_5)].

Phenylacetylene (153.1 mg, 1.5 mmol) was reacted with 1-iodo-2-nitrobenzene (249.0 mg, 1.00 mmol), NaOH (80.0 mg, 2.00 mmol) and γ-Fe_2_O_3_@PEG@PAMAM G_0_-Cu (0.8 mol%, 0.006 g) in water (5 mL) at 80 °C to yield 65% (145.0 mg) 1-nitro-2-(phenylethynyl)benzene (**1n**). Chromatography: *n*-hexane/EtOAc, 8:2. **^1^H-NMR** (400 MHz, CDCl_3_) *δ* 7.35-8.06 (m, 9H, Ar-H); **^13^C-NMR** (62.9 MHz, CDCl_3_) *δ* 87.5, 89.8, 120.2, 121.7, 123.0, 124.2, 125.5, 131.2, 131.8, 132.3, 134.5, 149.3.

Phenylacetylene (153.1 mg, 1.5 mmol) was reacted with 1-bromo-2-nitrobenzene (202.0 mg, 1.00 mmol), NaOH (80.0 mg, 2.00 mmol) and γ-Fe_2_O_3_@PEG@PAMAM G_0_-Cu (0.8 mol%, 0.006 g) in water (5 mL) at 80 °C to yield 45% (100.4 mg) 1-nitro-2-(phenylethynyl)benzene (**1n**). Chromatography: *n*-hexane/EtOAc, 8:2. **^1^H-NMR** (400 MHz, CDCl_3_) *δ* 7.35-8.08 (m, 9H, Ar-H); **^13^C-NMR** (62.9 MHz, CDCl_3_) *δ* 87.5, 89.9, 120.0, 121.4, 123.2, 124.2, 125.5, 131.5, 131.9, 132.3, 134.5, 149.1.

**3-(phenylethynyl)pyridine (1o)**. This compound is known in the literature [[1](#_ENREF_1)].

3-ethynylpyridine (154.1 mg, 1.5 mmol) was reacted with iodobenzene (204.0 mg, 1.00 mmol), NaOH (80.0 mg, 2.00 mmol) and γ-Fe_2_O_3_@PEG@PAMAM G_0_-Cu (0.8 mol%, 0.006 g) in water (5 mL) at 80 °C to yield 92% (164.8 mg) 1,2-diphenylacetylene (**1a**). Chromatography: *n*-hexane/EtOAc, 8:2. **^1^H-NMR** (400 MHz, CDCl_3_) *δ* 7.30-7.48 (m, 5H, Ar-H), 8.77-9.02 (m, 4H, Ar-H); **^13^C-NMR** (62. MHz, CDCl_3_) *δ* 82.5, 96.2, 119.5, 121.8, 128.9, 128.6, 129.0, 129.2, 129.5, 131.6, 139.2, 156.8, 158.6.

3-ethynylpyridine (154.1 mg, 1.5 mmol) was reacted with bromobenzene (157.0 mg, 1.00 mmol), NaOH (80.0 mg, 2.00 mmol) and γ-Fe_2_O_3_@PEG@PAMAM G_0_-Cu (0.8 mol%, 0.006 g) in water (5 mL) at 80 °C to yield 88% (157.7 mg) 1,2-diphenylacetylene (**1a**). Chromatography: *n*-hexane/EtOAc, 8:2. **^1^H-NMR** (250 MHz, CDCl_3_) *δ* *δ* 7.30-7.48 (m, 5H, Ar-H), 8.77-9.04 (m, 4H, Ar-H); **^13^C-NMR** (62. MHz, CDCl_3_) *δ* 82.4, 96.1, 119.3, 121.9, 128.7, 128.5, 129.0, 129.3, 129.5, 131.5, 139.1, 156.8, 158.6.

**5-(phenylethynyl)pyrimidine** (**1p**). This compound is known in the literature [[1](#_ENREF_1)].

5-ethynylpyrimidine (156.1 mg, 1.5 mmol) was reacted with iodobenzene (204.0 mg, 1.00 mmol), NaOH (80.0 mg, 2.00 mmol) and γ-Fe_2_O_3_@PEG@PAMAM G_0_-Cu (0.8 mol%, 0.006 g) in water (5 mL) at 80 °C to yield 91% (163.9 mg) 1,2-diphenylacetylene (**1a**). Chromatography: *n*-hexane/EtOAc, 8:2. **^1^H-NMR** (400 MHz, CDCl_3_) *δ* 7.29-7.48 (m, 5H, Ar-H), 8.78 (s, 2H, Ar-H), 9.08 (s, 1H, Ar-H); **^13^C-NMR** (62.9 MHz, CDCl_3_) *δ* 82.3, 96.3, 119.8, 121.6, 128.5, 128.8, 129.2, 129.3, 131.6, 139.2, 156.6, 158.4.

5-ethynylpyrimidine (156.1 mg, 1.5 mmol) was reacted with bromobenzene (157.0 mg, 1.00 mmol), NaOH (80.0 mg, 2.00 mmol) and γ-Fe_2_O_3_@PEG@PAMAM G_0_-Cu (0.8 mol%, 0.006 g) in water (5 mL) at 80 °C to yield 85% (153.1 mg) 1,2-diphenylacetylene (**1a**). Chromatography: *n*-hexane/EtOAc, 8:2. **^1^H-NMR** (400 MHz, CDCl_3_) *δ* 7.29-7.49 (m, 5H, Ar-H), 8.78 (s, 2H, Ar-H), 9.07 (s, 1H, Ar-H); **^13^C-NMR** (62.9 MHz, CDCl_3_) *δ* 82.2, 96.3, 119.8, 121.5, 128.5, 128.7, 129.2, 129.4, 131.4, 139.2, 156.6, 158.3.

**References**

[1] M. Kazemnejadi, Z. Rezazadeh, M.A. Nasseri, A. Allahresani, M. Esmaeilpour, Imidazolium chloride-Co (iii) complex immobilized on Fe_3_O_4_@SiO_2_ as a highly active bifunctional nanocatalyst for the copper-, phosphine-, and base-free Heck and Sonogashira reactions, Green Chemistry, 21 (2019) 1718-1734.

[2] A.R. Sardarian, M. Kazemnejadi, M. Esmaeilpour, Bis-salophen palladium complex immobilized on Fe_3_O_4_@SiO_2_ nanoparticles as a highly active and durable phosphine-free catalyst for Heck and copper-free Sonogashira coupling reactions, Dalton Transactions, 48 (2019) 3132-3145.

[3] S. Yu, J. Wu, X. He, Y. Shang, Ferrocenyl bisoxazoline as an efficient non‐phosphorus ligand for palladium‐catalyzed copper‐free Sonogashira reaction in aqueous solution, Applied Organometallic Chemistry, 32 (2018) e4156.

[4] A.S. Roy, J. Mondal, B. Banerjee, P. Mondal, A. Bhaumik, S.M. Islam, Pd-grafted porous metal–organic framework material as an efficient and reusable heterogeneous catalyst for C–C coupling reactions in water, Applied Catalysis A: General, 469 (2014) 320-327.

[5] M.A. Nasseri, Z. Rezazadeh, M. Kazemnejadi, A. Allahresani, A Co–Cu bimetallic magnetic nanocatalyst with synergistic and bifunctional performance for the base-free Suzuki, Sonogashira, and C–N cross-coupling reactions in water, Dalton Transactions, 49 (2020) 10645-10660.
